# Protective Effects Oncorneal Endothelium During Intracameral Irrigation Using N-(2)-l-alanyl-l-Glutamine

**DOI:** 10.3389/fphar.2020.00369

**Published:** 2020-03-27

**Authors:** Mengyi Jin, Yanzi Wang, Yixin Wang, Yunpeng Li, Guoliang Wang, Xuezhi Liu, Yuhua Xue, Zuguo Liu, Cheng Li

**Affiliations:** ^1^Eye Institute & Affiliated Xiamen Eye Center, School of Medicine, Xiamen University, Xiamen, China; ^2^Fujian Provincial Key Laboratory of Ophthalmology and Visual Science, Xiamen, China; ^3^School of Pharmaceutical Sciences, Xiamen University, Xiamen, China

**Keywords:** N (2)-L-Alanyl-glutamine, corneal endothelium, intracameral irrigation, mitochondria, ocular hypertension

## Abstract

Corneal endothelial disease is a global sight-threatening disease, and corneal transplantation using donor corneas remains the sole therapeutic option. A previous work demonstrated that N (2)-alanyl-glutamine (Ala-Gln) protected against apoptosis and cellular stress, and maintained intestinal tissue integrity. In this pursuit, the present study aimed to examine the effect of Ala-Gln in the protection of the corneal endothelium and expand its range of potential clinical applications. Mice in the control group were intracamerally irrigated with Ringers lactate injection, whereas those in the experimental group were irrigated with Ringers lactate injection containing Ala-Gln. The mean intraocular pressure increased to 44 ± 3.5 mm Hg during intracameral irrigation (normal range 10.2 ± 0.4 mmHg). *In vivo* confocal microscopy results showed that the addition of Ala-Gln protected the morphology, structure, and density of the corneal endothelial cells. Optical Coherence Tomography (OCT) measurements showed that corneal thickness was not significantly different between the two groups, because of the immediate corneal edema after irrigation, but the addition of Ala-Gln obviously promoted the recovery of the corneal edema. Scanning electron microscopy indicated that the corneal endothelial cells were severely ruptured and exfoliated in the Ringer’s group accompanied with cellular edema, when compared with the Ala-Gln group. The intracameral irrigation using Ala-Gln protected the structure and expression of cytoskeleton and Na-K-ATPase, which exhibited a regular distribution and significantly increased expression in comparison to Ringer’s group. Furthermore, Ala-Gln maintained the mitochondrial morphology and increased the activity of mitochondria. Moreover, transmission electron microscopy showed that intracameral irrigation of Ala-Gln reversed the ultrastructural changes induced by the acute ocular hypertension in mice. Our study demonstrates that the intracameral irrigation of Ala-Gln effectively maintained the corneal endothelial pump function and barrier function by protecting the mitochondrial function and preventing the rearrangement of cytoskeleton in acute ocular hypertension in mice.

## Introduction

The corneal endothelium located at the Descemet’s membrane, is a regular arrangement of hexagonal cells, which maintains the corneal transparency through stromal hydration, and acts as a barrier between the stroma and the anterior chamber (Riley et al., 1998; Bourne, 2003; Fischbarg and Maurice, 2004). The corneal endothelium is responsible for pumping fluid out of the corneal stroma to prevent the development of edematous haze. Unlike the corneal epithelium, human corneal endothelial cells exhibit a limited proliferation potential. Hence, an excessive decrease in the endothelial cell density can result from surgeries or accidental trauma, which can induce the damage of barrier and pump functions, and lead to an irreversible corneal edema, haze, and eventually loss of visual acuity (Joyce, 2003). Fuchs’ endothelial dystrophy, Bullous keratopathy, a degenerative aging change of the endothelium and corneal decompensation after surgery are the common corneal endothelial diseases (Soh et al., 2017). At present, the only therapeutic option for corneal endothelial diseases is corneal transplantation using donor corneas (Tan et al., 2012). Although the introduction of new transplantation techniques, such as Descemet’s membrane endothelial Keratoplasty, have decreased the risk of corneal transplantation techniques (Melles et al., 2008; Price and Price, 2010), there are still problems in the surgery, such as technical difficulties in surgery, graft rejection, cell loss, and donor corneal shortage. Consequently, in order to protect the corneal endothelium effectively, there is an urgent need to investigate novel and sustainable treatments.

Glutamine is the most abundant amino acid in the blood (Calder, 1994), and is essential in various metabolic processes, especially in the intestine. The intestine utilizes glutamine for the maintenance of the barrier function and in the regulation of the inflammation and apoptosis (Carneiro et al., 2006; Li and Neu, 2009; Hou et al., 2013). Glutamine is very poorly soluble and unstable during storage and heat sterilization, which limits its addition in the existing preparations (Stehle et al., 1984). However, these shortcomings can be overcome by employing a synthetic dipeptide containing glutamine, L-alanyl-L-glutamine (Ala-Gln) (Furst et al., 1987; Furst et al., 1990).

Ala-Gln supplementation was found to reduce the release of inflammatory factors, attenuate the inflammatory response, increase proliferation, and prevent apoptosis and oxidative injury under different physiological conditions in a similar manner to that of glutamine (Haynes et al., 2009; Cunha Filho et al., 2011; Ueno et al., 2011; Nosworthy et al., 2016). Several studies have showed the protective effects of Ala-Gln against the injury induced by myocardial, intestinal, hepatic or cerebral ischemia reperfusion (Tazuke et al., 2003; Araujo Junior et al., 2011; Vasconcelos et al., 2015; Liu et al., 2018). In addition, Ala-Gln is usually used in enteral nutrition. Clinical and experimental investigations demonstrated that Ala-Gln increased the expression of tight junction proteins and maintained the intestinal mucosal thickness and villus height to preserve the gut barrier function (Liu et al., 1997; Zhang et al., 2019). Although Ala-Gln is widely used in various conditions, so far, it has no application reports in the field of ophthalmology. Considering the protective effect of Ala-Gln in the ischemia reperfusion and gut barrier function, in this study, we explored the protective effects and mechanism of L-alanyl-L-glutamine on the corneal endothelium by intracameral irrigation in acute ocular hypertension.

## Materials and Methods

### Chemicals and Reagents

Ala-Gln (lot number: YH0170926) was purchased from Shanghai Yihe Biotechnology Co., Ltd (Shanghai, China; purity > 99%). Sodium Lactate Ringer’s Injection was from Zhejiang Tianrui Pharmaceutical Co., Ltd (Wenzhou, China). Alexa Fluor 555 Phalloidin (catalog no. A34055, Invitrogen, California, USA), Anti-Sodium Potassium ATPase antibody (catalog no. 76020, Abcam, Cambridge, UK), Alexa Fluor 594-conjugated donkey anti-rabbit IgG (catalog no. A21207, Invitrogen, California, USA) and 4′,6-diamidino-2-phenylindole (DAPI; catalog no. H-1200, Vector, Burlingame, CA, USA) were purchased. MitoTracker Red CMXRos (catalog no. 40741ES50) was from Shanghai Yisheng Biological Technology Co., Ltd. (Shanghai, China).

### Animals

The Institute of Cancer Research (ICR) mice, aged 6-8weeks, were obtained from the Experimental Animal Centre of Xiamen University. All the animals were housed with free access to food and water. All experimental procedures conformed to the Association for Research in Vision and Ophthalmology (ARVO) for the use of animals in ophthalmic and vision research, and were approved by the Experimental Animal Ethics Committee, Xiamen University.

### Intracameral Irrigation Model

Fifteen mice were randomly divided into the Ringer’s group and Ala-Gln group. Acute high intraocular pressure has a transient deleterious effect on the structure and function of corneal endothelial cells (Li et al., 2017). Before high intraocular pressure was induced, the mice were anesthetized with 50 mg/kg bodyweight of pentobarbital through intraperitoneal injection. After maintaining pupil dilation with Tropicamide (Akorn, Inc., Lake Forest, IL, USA) and achieving corneal analgesia using eye drops containing 0.5% proparacaine hydrochloride ophthalmic solution (Alcaine; Alcon Laboratories, Inc., Fort Worth, TX), a clear corneal tunnel was performed on the nasal side of the right eye with a 32-gauge needle (Cat. no. PRE-32013P, TSK Laboratories, Japan). Subsequently, a 34 g micro syringe needle (Cat. no. 207434, Hamilton) was introduced into the anterior chamber through the corneal tunnel, thereby avoiding the contact with the corneal endothelium and the lens. The needle was connected to a 500 ml container of Sodium Lactate Ringer’s Injection or irrigation solution containing 5 mM Ala-Gln for the anterior chamber perfusion. The concentration of Ala-Gln was selected according to previous studies (Haynes et al., 2009; Raspe et al., 2013). The mice were randomized during the perfusion with either Sodium Lactate Ringer’s Injection or Sodium Lactate Ringer’s Injection containing 5 mmol/L Ala-Gln for 60 minutes. Intraocular pressure was increased and maintained at 44 ± 3.5 mm Hg by elevating the solution container. At days 0, 2 and 10 after termination the perfusion, all mice were subjected to examination, followed with euthanization and histological examination was conducted.

### *In Vivo* Confocal Microscopic Studies (IVCM)

Images of the corneal endothelium and stroma were collected using the Heidelberg Retinal Tomography (HRT3)/Rostock Cornea Module (RCM) (Heidelberg Engineering Inc., Germany) *in vivo* as described previously (Trinh et al., 2008). After the mice were anesthetized, a drop of carbomer gel was applied to the lens cap as a coupling medium. The central cornea was maintained in contact with the cap. By controlling the depth of the z-direction at 2 µm increments manually, the representative images from superficial epithelium to the corneal endothelium were recorded. Corneal endothelial density in cells/mm^2^ was analyzed by the procedure associated with the HRT3/RCM after collecting the corneal endothelial images.

### Optical Coherence Tomography (OCT) Studies

OCT (RT-100, Optovue Inc., Fremont, CA, USA) was employed to obtain the corneal cross-section photographs for evaluating the central corneal thickness as described previously (Zhang et al., 2017). In brief, the eyes were washed three times with phosphate buffer solution (PBS), and mounted in front of the optical scanning probe to obtain the images, and the corneal thickness was analyzed using4 quadrant-scans.

### Whole Mounted Cornea Staining

Mice were sacrificed using anesthesia. The eyes were enucleated, and the corneas were cut along the limbus. Whole dissected corneas were instantly fixed using4% paraformaldehyde in PBS overnight at 4°C, and then fixed with cold acetone at -20 °C for 3 minutes. After washing three times with PBS containing 3% Triton X-100 and 1% dimethyl sulfoxide (TD buffer) (10 minutes per wash), to exclude the nonspecific labeling, each tissue was first incubated in 2% bovine serum albumin (BSA)/PBS for 60 minutes at room temperature (RT). For Phalloidin staining, tissues were incubated with Phalloidin (1:150) for 90 minutes at RT. For Sodium-Potassium-ATPase staining, samples were incubated overnight with polyclonal rabbit anti-sodium-potassium-ATPase (1:500) in a mixture of 1% BSA in 3% TD Buffer at 4 °C. After washing with PBS three times, the tissues were incubated with Alexa Fluor 594-conjugated donkey anti-rabbit IgG (1:200) in the dark for 5 hours at 4 °C. After three washes in PBS, the corneal endothelium was mounted up on the glass slide and counterstained with DAPI. Finally, the digital photographs of the representative areas were taken using a laser confocal scanning microscope (Olympus FV1000MPE-B, Olympus).

### MitoTracker Red (MTR) Staining

MTR is a fluorescent probe widely used to monitor the changes in mitochondria and quantify the number of mitochondria (Poot et al., 1996; Gilson et al., 2003; Sansanwal et al., 2010). According to the manufacturer’s instructions, the eyes were enucleated and placed in serum free Dulbecco Modified Eagle Medium, and the corneas were cut along the limbus. The whole dissected corneas were immediately immersed in the Dulbecco Modified Eagle Medium containing 250 nM MitoTracker probe, and incubated in the dark for 40 min at 37 °C. Subsequently, the corneas were washed three times and mounted up on the glass slide and counterstained with DAPI. Finally, the mitochondria in the corneal endothelium were viewed under the confocal microscope (Olympus FV1000MPE-B). The mean intensity of staining was measured by ImageJ software (US National Institutes of Health, Bethesda, MD).

### Transmission Electron Microscopy (TEM) Studies

Sample preparation for TEM were performed according to method described previously (White et al., 2017). Briefly, the corneas were harvested and fixed immediately in 0.1M phosphate buffer containing 2.5% glutaraldehyde at 4°C and washed three times in PBS before post-fixing in 1% osmium tetroxide. Subsequently, the samples were dehydrated in 30% and 50% ethanol and stained with uranyl acetate in 70% ethanol, followed by dehydration with a graded ethanol series. Subsequently, the samples were embedded in resin, and cut into ultrathin sections (70 nm) followed by staining with lead citrate. Sections were examined and photographed at 80 kV with a transmission electron microscope (HT-7800, HITACHI, Japan).

### Scanning Electron Microscopy (SEM) Studies

The fresh corneas were fixed overnight in 0.1M phosphate buffer containing 2.5% glutaraldehyde at 4° C, followed by dehydration using a graded ethanol series. Subsequently, the samples were dried to a critical point and sputter-coated with a layer of gold before imaging with a scanning electron microscopy (JSM-6390LV; JEOL, Tokyo, Japan).

### Statistical Analysis

All experiments were repeated at least three times unless otherwise indicated. The statistical results are presented as mean ± SEM, and analyzed by an unpaired Student’s t-test with graphing software (GraphPad Prism 7.0; GraphPad Software, San Diego, CA, USA). A value of P < 0.05 was defined as statistically significant.

## Results

### Ala-Gln Protected the Morphology and Density of Corneal Endothelial Cells

**Figure 1:**
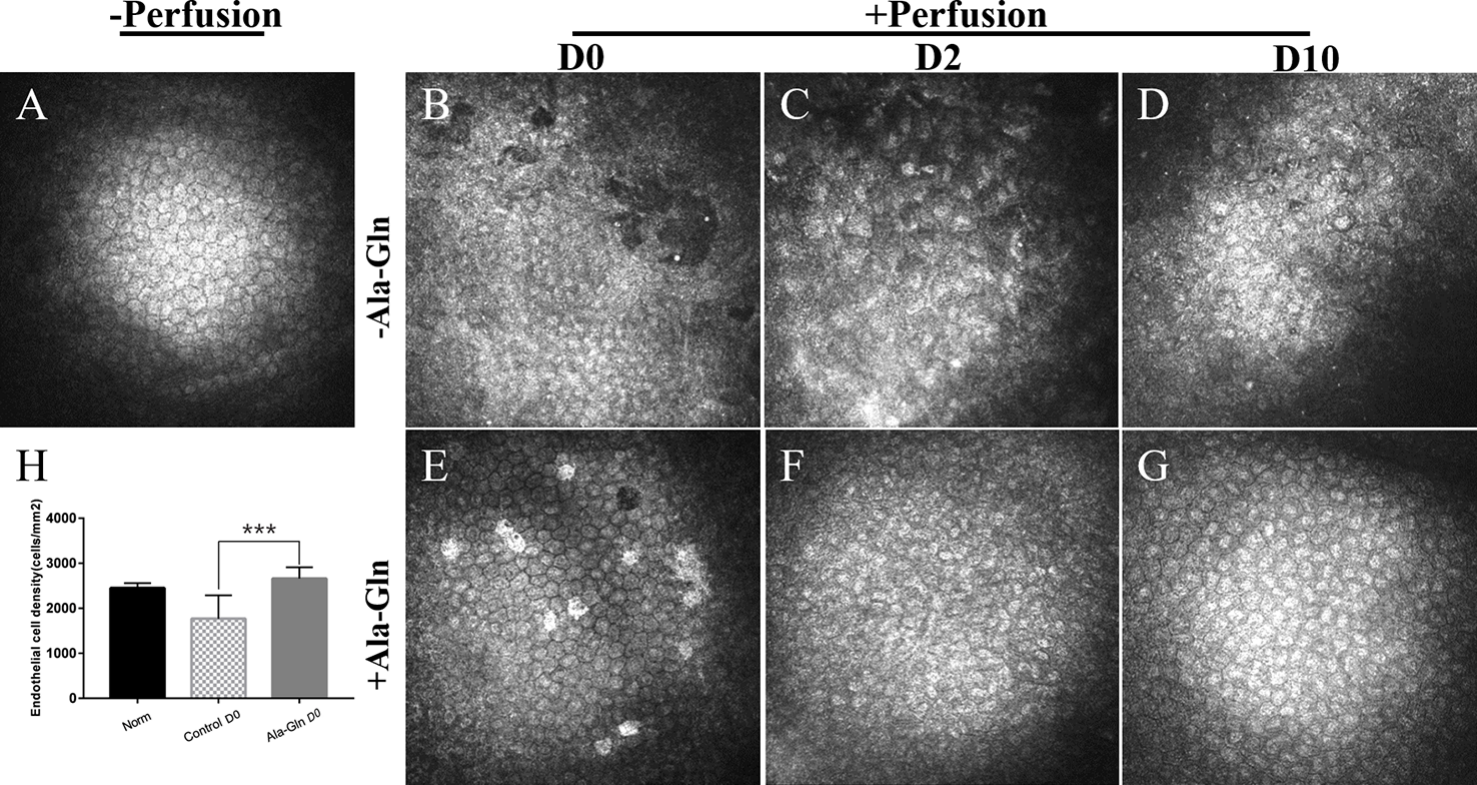
Evaluation of the morphology and density of corneal endothelial cells by IVCM. Representative images demonstrate the morphology and structure of corneal endothelial cells pre-irrigation **(A)** and post-irrigation in the Ringer’s **(B–D)** or Ala-Gln **(E–G)** group at different time points. **(H)** Changes in the average endothelial cell density. Data are represented as mean ± SEM. ***P < 0.001. (n = 6/group).

IVCM is a noninvasive technique that evaluates the appearance and structure of the endothelial cells *in-situ* in a narrow region of the cornea. Before irrigation, the corneal endothelial cells demonstrated a characteristic morphology with a regular hexagonal appearance, cell density of 2453 ± 103.2 cells/mm^2^ (**Figure 1A**). After irrigation, the endothelium in the Ringer’s group was damaged and irregular in appearance with some endothelial cells lost and some gaps or holes were observed (**Figure 1B**). Furthermore, the corneal endothelial density was significantly reduced (1807 ± 463.3cells/mm^2^) in comparison to normal endothelium. This injury was significant attenuated when Ala-Gln was added in the Ringer’s solution, showing a cell density of 2774.4 ± 276.7 cells/mm^2^ (**Figure 1E**). The same mice were assessed by IVCM at day 2 and 10 to evaluate whether there was further loss of endothelial cell. At day 2, the endothelial cells in the Ringer’s group distributed even more pleomorphism. Moreover, the average endothelial cell size appeared to have increased, compared with normal and Ala-Gln cornea and presented as bullous keratopathy (**Figure 1C**). The cell density was further reduced to1464 ± 448.5 cells/mm^2^. Whereas Ala-Gln corneal endothelial cells keep the normal size and density (**Figure 1F**). At 10 days, the endothelial cells in Ringer’s group were still irregular and multiform (**Figure 1D**). Corneal endothelial cell morphology in the Ala-Gln group exhibited the normal appearance and was observed with a regular arrangement of hexagonal pattern at 10 days (**Figure 1G**). Statistical analysis conformed that the endothelial cell density in Ringer’s group dramatically decreased at **day 0** as compared with that in the normal endothelium, while it significantly increased in Ala-Gln treated groups compared with Ringer’s group (**Figure 1H**) Ala-Gln promoted the recovery of the corneal edema.

**Figure 2:**
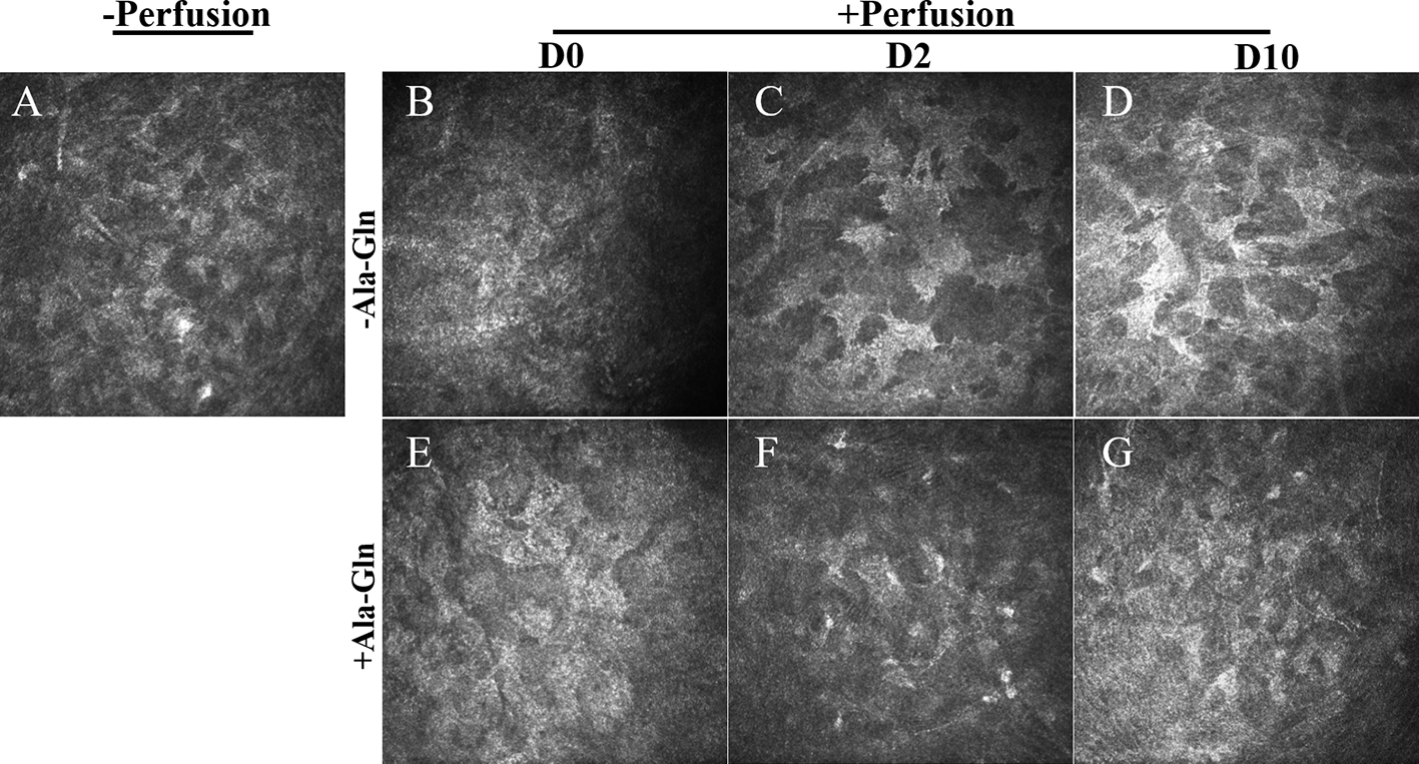
Assessment of the morphology of corneal stroma using IVCM. Representative images demonstrate the morphology of corneal stroma pre-irrigation **(A)** and post-irrigation in the Ringer’s **(B–D)** or Ala-Gln **(E–G)** group at different time points.

Compared with normal group (**Figure 2A**), in Ringer’s group, a hyper-reflective cytoplasm and extracellular lacunae with a honeycomb pattern was observed at, both the anterior and posterior stromal level of the middle cornea that persisted at least ten days after intracameral irrigation (**Figures 2B–D**). This honeycomb pattern was probably induced by focal corneal stroma edema or by syncytial cell bodies of the activated keratocytes. Stromal edema was apparent at day 0 (**Figure 2E**). However, this honeycomb pattern disappeared at day 2 with the addition of Ala-Gln with intracameral irrigation (**Figure 2E**) and the corneal stroma exhibited a normal appearance at day 10 (**Figure 2G**), which revealed that the addition of Ala-Gln during intracameral irrigation greatly promoted the recovery of the corneal edema.

### Ala-Gln Restored the Central Corneal Thickness

**Figure 3:**
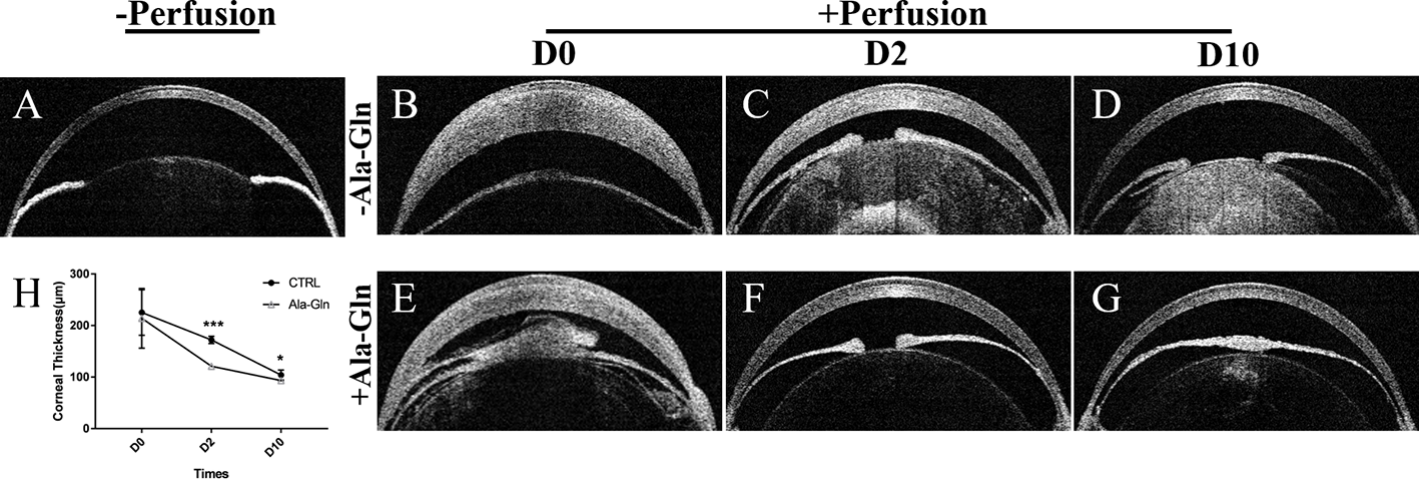
Evaluation of the central corneal thickness by OCT. Representative images demonstrate the central corneal thickness pre-irrigation **(A)** and post-irrigation in the Ringer’s **(B–D)** or Ala-Gln **(E–G)** group at different time points. **(H)** Comparative analysis shows an increased central corneal thickness in the Ringer’s group than the Ala-Gln group at different time points after irrigation. Data are represented as mean ± SEM. *P < 0.05, ***P < 0.001. (n = 3/group).

Corneal thickness is closely related to the endothelial barrier and pump functions (Mergler and Pleyer, 2007). After IVCM showed the presence of corneal stromal edema, to validate these findings, OCT was employed to evaluate the thickness of the central cornea. It was found that the corneal thickness was not significantly different between the two groups immediately after irrigation, because of the corneal edema caused by acute ocular hypertension (**Figures 3B, E**). Compared with Ringer's group (**Figure 3C**), the increase in corneal thickness due to corneal edema was significantly weakened at day 2 (**Figure 3F**), which indicated the addition of Ala-Gln greatly promoted the recovery of the corneal edema and probably had greater barrier function and pump function in comparison to the Ringer’s group. At day 10, there was no statistical difference in corneal thickness between the Ala-Gln and the normal cornea (**Figures 3A, G**), while the corneal thickness in the Ringer’s group remained lightly elevated compared to Ala-Gln group (**Figure 3D**). The statistical analysis of corneal thickness at day 0, day 2 and day 10 is presented in **Figure 3H**.

### Ala-Gln Reversed the Cytoskeleton Distribution

**Figure 4:**
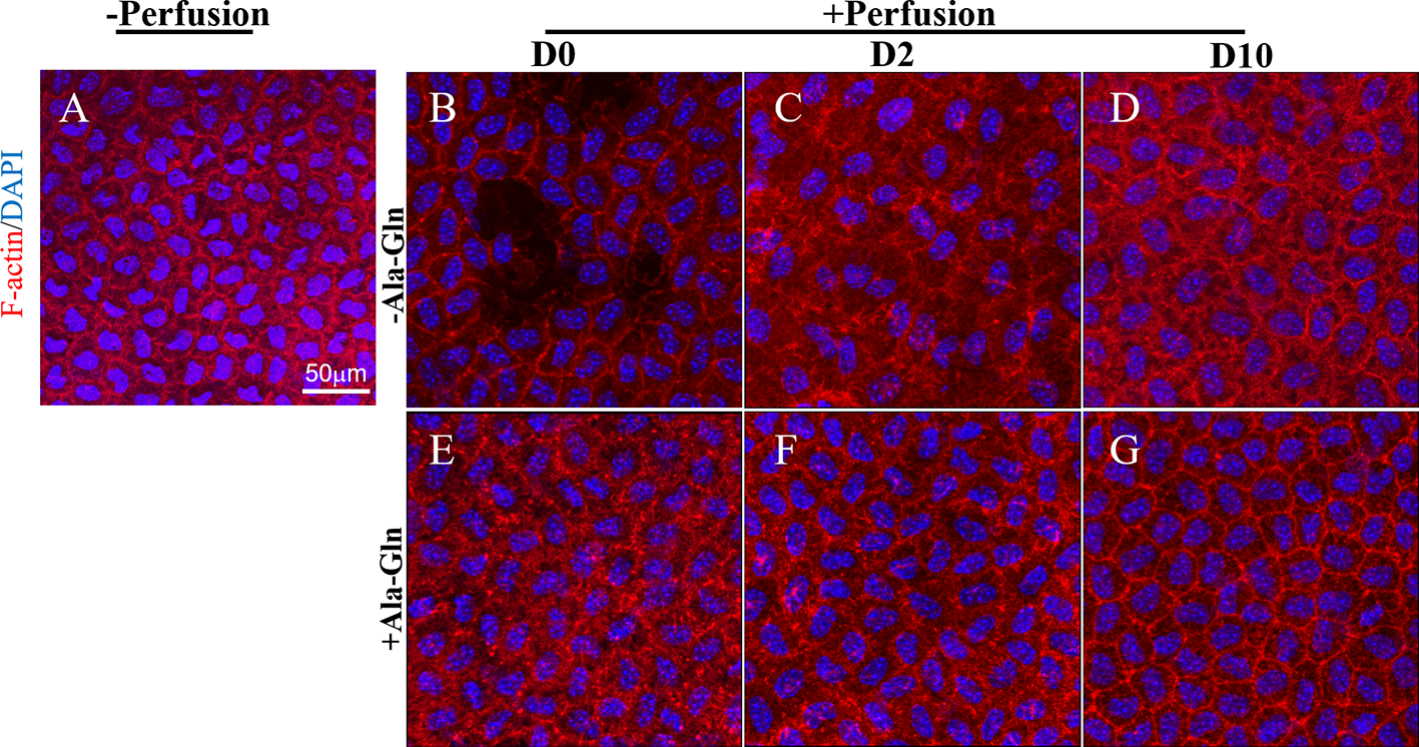
Blockage of the changes of F-actin distribution and cytoskeleton rearrangement by Ala-Gln induced by intracameral irrigation. Representative images of F-actin (red) immunofluorescent staining in corneal endothelium pre-irrigation **(A)** and post-irrigation in the Ringer’s **(B–D)** or Ala-Gln **(E–G)** group, at different time points. Nuclei were stained with DAPI (blue).

F-actin cytoskeleton distribution was detected using immunofluorescence with Texas Red–X phalloidin. F-actin microfilaments were confined to the boundary of the apical cells and composed the double-track appearance under normal conditions (**Figure 4A**). Intracameral irrigation caused the disappearance of the circumferential appearance and a rearrangement of the F-actin cytoskeletal organization. Following intracameral irrigation, the corneas exhibited a scattered loss of the endothelial cells and gross endothelial sloughing (**Figure 4B**). At day 2, the expression was diffused throughout the cytoplasm (**Figure 4C**). Ten days after the intracameral irrigation, the F-actin cytoskeleton partially underwent reorganization, and its distribution was partially recovered (**Figure 4D**). This alteration was almost reversed by Ala-Gln (**Figures 4E–G**).

### Ala-Gln Enhanced the Pump Function of Corneal Endothelium

**Figure 5:**
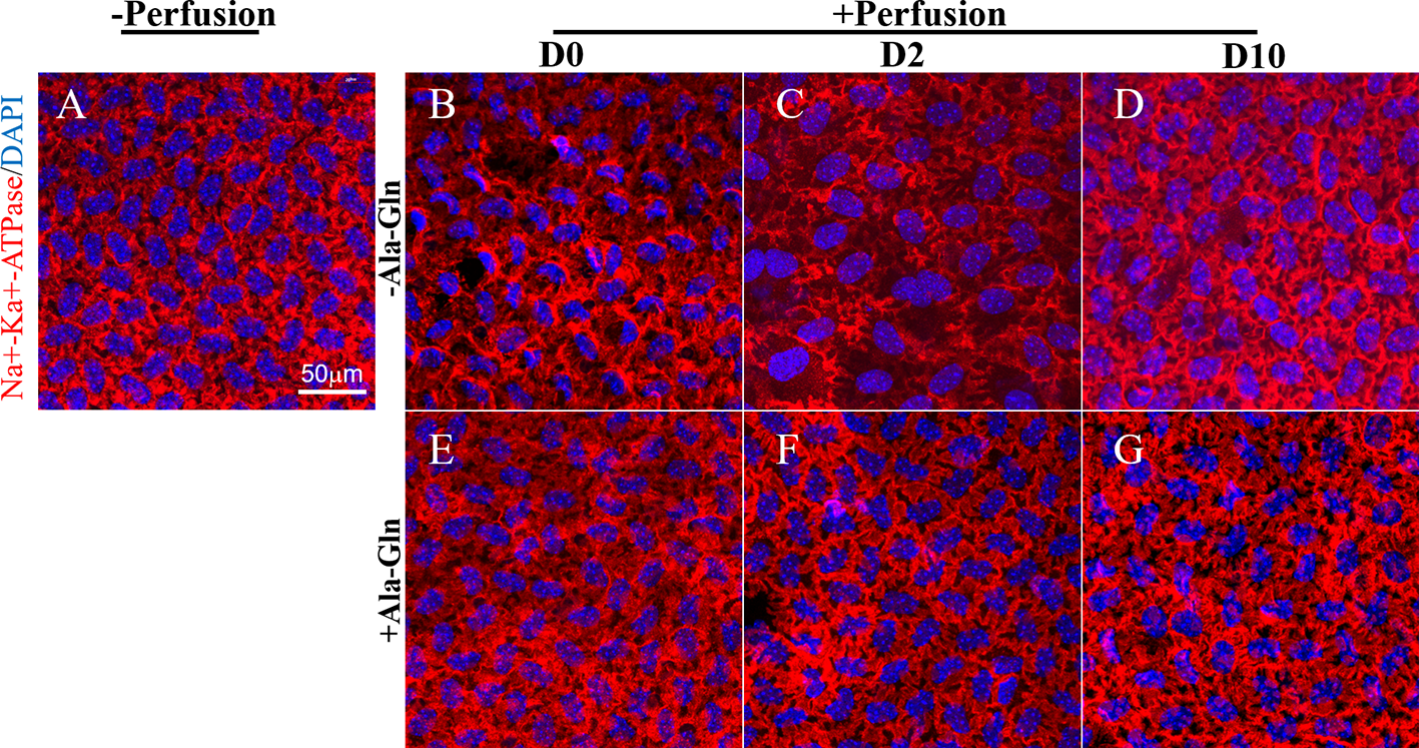
Blockage of the disruption of Na-K-ATPase location by Ala-Gln induced by ocular hypertension and its enhanced expression. Representative images of Na-K-ATPase (red) immunofluorescent staining in corneal endothelium pre-irrigation **(A)** and post-irrigation in the Ringer’s **(B–D)** or Ala-Gln **(E–G)** group at different time points. Nuclei were stained with DAPI (blue).

The Na-K-ATPase is accountable for the regulation of the pump functions of the corneal endothelium, and localizes in the basolateral membrane in the corneal endothelium (Fischbarg and Lim, 1974; Tervo et al., 1977). Na-K-ATPase is expressed around the cell membrane evenly and continuously (**Figure 5A**). Following intracameral irrigation, we found that its expression was disrupted and scattered (**Figure 5B**). At day 2, the Na-K-ATPase expression was scattered and removed from the basolateral membrane (**Figure 5C**). Ten days after intracameral irrigation, the recovery was still incomplete (**Figure 5D**). In contrast to the sparse staining, intracameral irrigation with Ala-Gln increased the expression of Na-K-ATPase and restored its localization (**Figures 5E, F**). At day 10, the expression was not significantly different than the normal levels, and it expressed around the cell membrane continuously (**Figure 5G**).

### Ala-Gln Maintained the Corneal Endothelial Barrier Function and Cell Junction Integrity

**Figure 6:**
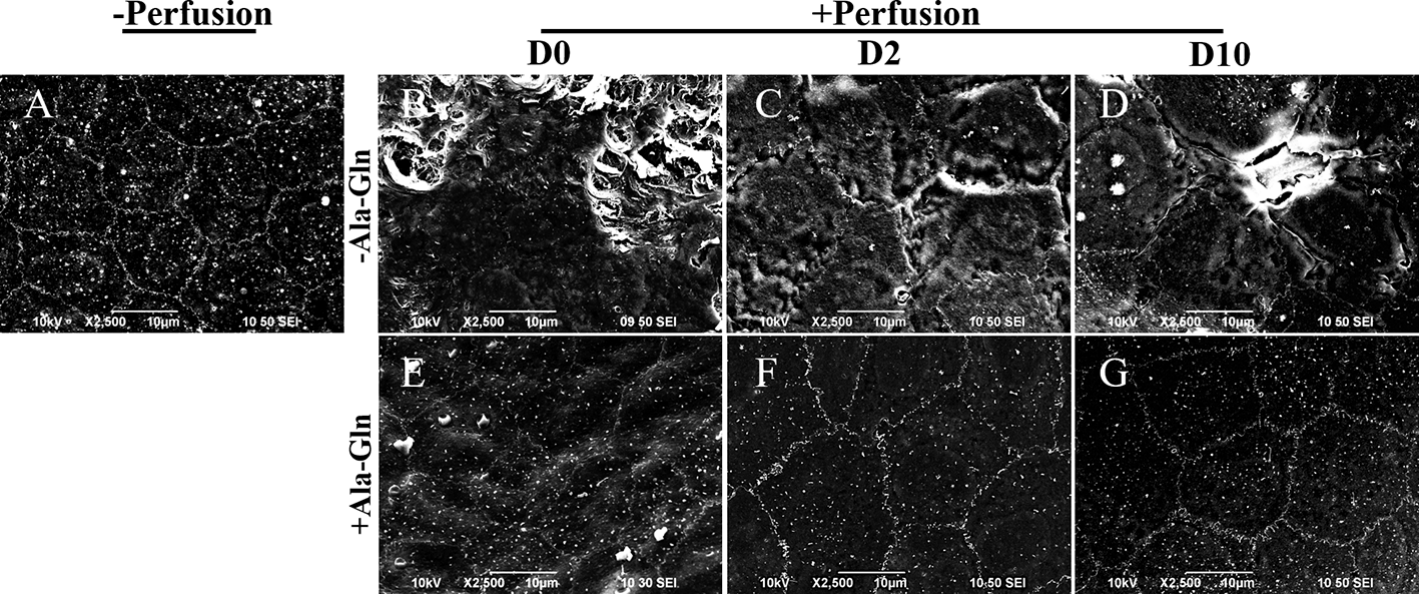
Scanning electron micrographs of the partial surface of the corneal endothelium. Representative images of scanning electron microscopy in corneal endothelium pre-irrigation **(A)** and post-irrigation in the Ringer’s **(B–D)** or Ala-Gln **(E–G)** group at different time points.

Scanning electron microscopy showed that normal endothelium maintained a hexagonal appearance and a continuous flat layer (**Figure 6A**), however, the intracameral irrigation caused the extensive rupture of corneal endothelial cells, and the cell junctions were partially disrupted (**Figure 6B**). At day 2, the corneal endothelial cells in Ringer’s group were swollen, and the intercellular functions were widened (**Figure 6C**). Ten days later, the cell junctions in Ringer’s group were still poorly formed (**Figure 6D**), whereas the endothelium in the Ala-Gln group showed an undisrupted monolayer and intact intercellular junctions (**Figures 6E, F**). In addition, the cells exhibited a regular and hexagonal appearance with abundant microvilli (**Figure 6G**). The results demonstrate that the addition of Ala-Gln during irrigation helps to maintain the corneal endothelial barrier function and cell junction integrity.

### Ala-Gln Improved the Activity of Mitochondria

**Figure 7:**
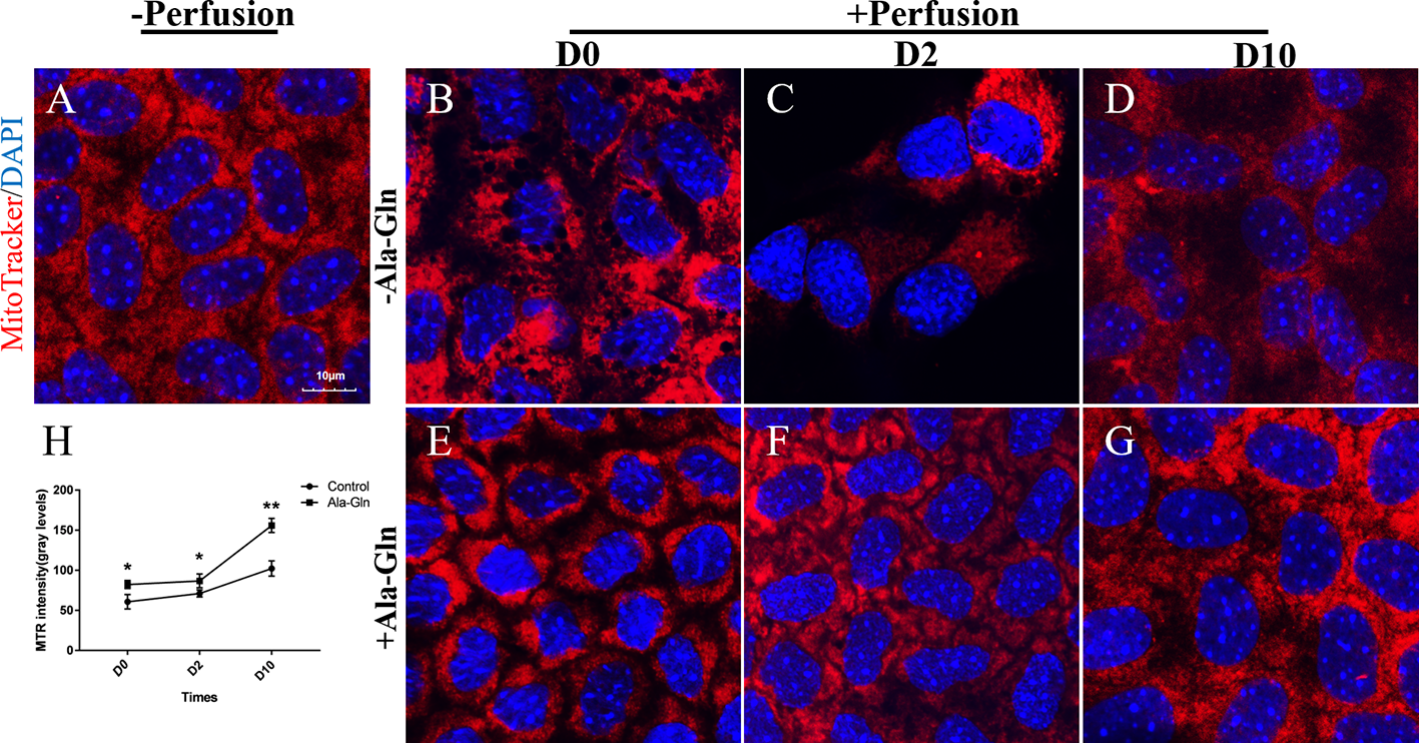
Prevention of the decrease in the mitochondrial activity by Ala-Gln caused by intracameral irrigation. Representative images of MitoTracker (red) fluorescent staining in corneal endothelium pre-irrigation **(A)** and post-irrigation in the Ringer’s **(B–D)** or Ala-Gln **(E–G)** group at different time points.Nuclei were stained with DAPI (blue). **(H)** Relative fluorescence intensity of the mitochondria in corneal endothelium. Data are represented as mean ± SEM. *P < 0.05, **P < 0.01. (n = 3/group).

To detect the effect of intracameral irrigation on the morphology and activity of mitochondria, the mitochondria of living cells were stained with specific fluorescence staining using MTR. Normal endothelial cells existed intense staining and intact mitochondrial network (**Figure 7A**). However, after intracameral irrigation, the deteriorated fragmented mitochondrial structures were observed in Ringer’s group (**Figure 7B**). Particularly at day 2, the activity of mitochondria was significantly reduced while the corneal endothelium was presented as bullous keratopathy (**Figure 7C**). Compared with Ringer's group, addition of Ala-Gln reduced mitochondrial fragmentation caused by intracameral irrigation (**Figures 7E, F**). Ten days later, the MTR staining of Ringer’s group was still only weak and focal, whereas staining in Ala-Gln group reverted to normal expression (**Figures 7D, G**). The intensity of the staining was analyzed, and a statistically significant decrease was observed in the Ringer’s corneal endothelium compared to the corneal endothelium irrigated with the addition of Ala-Gln at day 0, day 2 and day 10 (**Figure 7H**). These results indicate that the Ala-Gln group exhibited strong MTR staining, and the addition of Ala-Gln preserved the activity of mitochondria during the acute ocular hypertension due to intracameral irrigation in mice.

### Ala-Gln Preserved the Structure of Mitochondria

**Figure 8:**
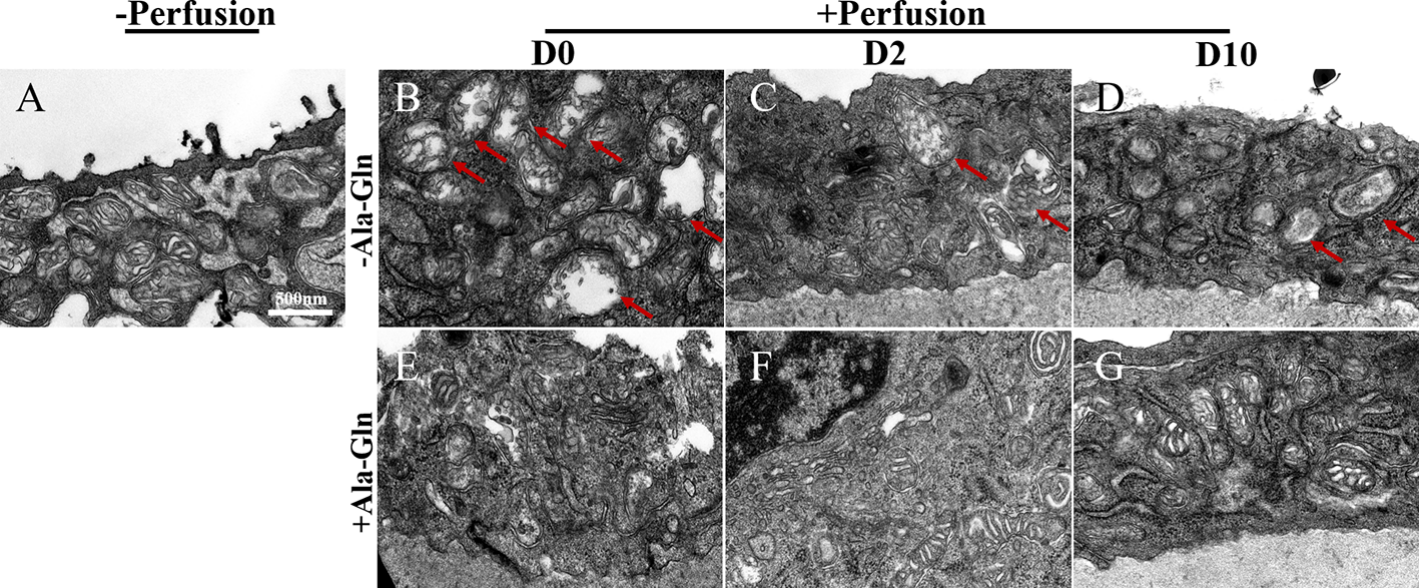
Transmission electron micrographs of the ultrastructural changes in corneal endothelium. Representative images of transmission electron microscopy in corneal endothelial cells pre-irrigation **(A)** and post-irrigation in the Ringer’s **(B–D)** or Ala-Gln **(E–G)** group at different time points. Note the swollen and diffusely degraded mitochondrial cristae (arrows).

Transmission electron microscopy can help to monitor the cellular ultrastructure. In the normal corneal endothelial cells, there was a high density of normal mitochondria (**Figure 8A**). Compared to the normal samples, after irrigation, the mitochondria in the Ringer’s group immediately displayed a large and distended morphology and the mitochondrial cristae were swollen and even degraded diffusely (**Figure 8B**). This appearance persisted for two more days (**Figure 8C**). In addition, the swollen and abnormal mitochondria were observed persistently till day 10 (**Figure 8D**). However, the cristae in the Ala-Gln group exhibited only a swollen morphology without degradation and expanded mitochondria (**Figure 8E**), and the mitochondria reverted to their normal shape after day 2 (**Figures 8F, G**).

## Discussion

Several clinical trials and experiments have demonstrated that glutamine is useful in improving the recovery of critically ill patients (Novak et al., 2002). Gln supplement is already a part of guidelines on enteral nutrition and for the parenteral nutrition in pancreatitis (Kreymann et al., 2006; Gianotti et al., 2009). In the intestine, Gln is known to activate numerous mitogen-activated protein kinases and growth factors that promotes the enterocyte proliferation, maintain multiple tight-junction proteins, such as claudin-1, occluding and ZO-1, regulate the NF-κB and STAT signaling to modulate the inflammatory pathways, and prevent apoptosis and cellular stress (Kim and Kim, 2017). Numerous researches were conducted to ascertain the role of Gln in the intestine; however, the role of Glnin the field of ophthalmology is much less evaluated. Considering its poor solubility and instability during heating sterilization and storage, our study utilized the synthetic dipeptide L-alanyl-L-glutamine, which was added to the irrigation solution.

The aim of this study was to detect the role of Ala-Gln in irrigation solutions, in an attempt to conclude whether it could protect the corneal endothelium. Our results revealed that Ala-Gln effectively prevented the corneal endothelium from the injury. After irrigation, there was an immediate significant increase in the corneal thickness because of the corneal edema. However, the decreased endothelial cell density, destructive barrier integrity, and pump function were reversed by Ala-Gln. Ten days later, the structure of corneal endothelium pretreated with Ala-Gln, and the expression of F-actin and Na-K-ATPase were nearly normalized. This effect of Ala-Gln was also accompanied by the restoration of mitochondrial function and structure caused by acute ocular hypertension intracameral irrigation. Thus, our results suggest that Ala-Gln blocks the irrigation-induced disruption of barrier function through the maintenance of the F-actin cytoskeleton and protection of the pump function in the corneal endothelium by preserving the structure and activity of the mitochondria.

It is reported that addition of glutamine increased the expression of ZO-1 and occludin in methotrexate-treated Caco-2 cells (Beutheu et al., 2013). Glutamine increased the claudin-1 expression to reduce IL-13-induced barrier dysfunction (Li et al., 2019). In stressed humans, glutamine is known to regulate the expression of tight junction proteins and cellular localization in Caco-2 cell monolayers to maintain the intestinal barrier function (Li et al., 2004). These studies suggest that Ala-Gln regulates the tight junction protein in corneal endothelial cells to preserve the barrier function. The tight junction and adhesion junction are closely associated with a circumferential bundle of actin microfilaments, which regulate the formation and maturation of the cell-cell adhesion. We found that Ala-Gln prevented the rearrangement of the F-actin cytoskeletal organization, and maintained a regular hexagonal cell pattern with smooth and distinct cell borders. We showed that Ala-Gln could inhibit the damage of the barrier function in the endothelial cells caused by acute ocular hypertension due to the intracameral irrigation, which was mediated by the prevention of the rearrangement of F-actin cytoskeleton.

Corneal endothelium is one of the most metabolically active tissues in body. The maintenance of the corneal hydration depends on main active transport (Na-K-ATPase activity) and multiple secondary membrane ion transporters (Geroski and Edelhauser, 1984; Bonanno, 2012). The powerful transport activity requires numerous mitochondria to satisfy the active metabolism (Bourne, 2003). These observations suggest that the metabolic therapies may represent a potential method to improve the corneal endothelium pump function and surgical outcomes. Gln plays an important role in the diverse metabolic processes. Its nitrogen molecules can be used as a substrate for the synthesis of nucleic acids while its 5-carbon skeleton can provide energy (Coster et al., 2004). We found that Ala-Gln reversed the down-regulation of Na-K-ATPase expression as well as the disappearance of the Na-K-ATPase from the basolateral membrane.

Mitochondria are the main source of cellular energy metabolism. Mitochondrial mass is decreased in the Fuchs endothelial corneal dystrophy, and the corneal endothelial cells subjected to oxidative stress (Halilovic et al., 2016). The abundance of mitochondria in the corneal endothelial cells is essential for maintaining corneal hydration and transparency. MTR is a fluorescent probe extensively utilized for viable staining of the mitochondria (Poot et al., 1996). MTR can be used to study the morphology and functional status of the mitochondria and quantify the number of mitochondria per cell (Gilson et al., 2003; Sansanwal et al., 2010). In an attempt to provide evidence that Ala-Gln protects mitochondrial activity and structure in the corneal endothelium, MTR analysis and transmission electron microscope was undertaken. It was found that Ala-Gln enhanced the activity of mitochondria and preserved the mitochondrial structure. Thus, Ala-Gln protected the structural and functional of mitochondria, and this protection contributed to the maintenance of corneal hydration and transparency.

One limitation of our research was the use of mice for the *in vivo* studies, as it was not possible to observe the long-term effects. We used corneal thickness and *in vivo* confocal microscopy as the dynamic parameters to show the barrier and pump function. After the intracameral irrigation, there was no statistical difference in the corneal thickness due to corneal edema caused by the mechanical damage and high intraocular pressure. However, the addition of Ala-Gln greatly promoted the recovery of corneal edema in 2 days. Corneal thickness and the morphology and density of the corneal endothelial cells returned to normal after ten days, while in the Ringer’s group, it remained significantly elevated. The result showed that Ala-Gln could maintain the corneal thickness and transparency by protecting the barrier and pump function.

Another limitation of our research was that the mechanism of Ala-Gln protection was not fully elucidated. Ala-Gln effectively maintained the corneal endothelial barrier pump function through by maintenance of the F-actin cytoskeleton and preservation of the structure and activity of mitochondria in the proposed mice model of acute ocular hypertension by intracameral irrigation. However, this model is far from enough to prove the protective effects of Ala-Gln for the corneal endothelium. Our research was limited to the effect on corneal endothelium, considering the effects of glutamine in the intestine and widely significance in other field. Whether Ala-Gln can also be used to protect other intraocular tissues which are damaged due to the oxidatively stress, still needs to be studied.

The most common etiology of corneal decompensation is intraocular surgery, especially the cataract surgery (Soh et al., 2017). In the cataract surgery, the most commonly used technique is phacoemulsification. However, the corneal injury associated with phacoemulsification is an important complication. This injury is caused by the excessive duration of the phacoemulsification, air bubbles, irrigation solutions, and mechanical damage. Corneal endothelial injury causes corneal edema, which can lead to irreversible bullous keratosis, in severe injury. The patients undergoing such surgeries may be at risk of blurred vision or even blindness in the advanced stages. Corneal tissue transplantation is the only treatment available presently, because of the lack of capacity to regenerate for this tissue. The results of the present research suggest that the addition of Ala-Gln in irrigation solutions may inhibit the destruction of the corneal endothelial barrier function and pump function caused by acute ocular hypertension, and may provide a new approach in the prevention of corneal endothelial damage.

## Data Availability Statement

All datasets generated for this study are included in the article/supplementary material.

## Ethics Statement

The animal study was reviewed and approved by Experimental Animal Ethics Committee of Xiamen University.

## Author Contributions

CL and ZL are responsible for the design and concept of the experiment. MJ, YaW, YiW, YL performed the experiments and made contribution to acquisition of date. GW, XL, YX contributed to statistical analysis. MJ drafted the manuscript and CL revised the manuscript critically and gave an interpretation of all the experiments.

## Funding

This study was supported in part by grants from the National Key R&D Program of China (2018YFA0107301), the National Natural Science Foundation of China (NSFC No. 81770891, 81870627, 81672955), and the Huaxia Translational Medicine Fund for Young Scholars (No. 2017‐A‐001).

## Conflict of Interest

The authors declare that the research was conducted in the absence of any commercial or financial relationships that could be construed as a potential conflict of interest.
